# Global mercury dataset with predicted methylmercury concentrations in seafoods during 1995–2022

**DOI:** 10.1038/s41597-025-04570-3

**Published:** 2025-02-11

**Authors:** Haifeng Zhou, Yumeng Li, Qiumeng Zhong, Xiaohui Wu, Sai Liang

**Affiliations:** 1 School of Environment, Beijing Normal University, Beijing, 100875 P. R. China; 2 Guangdong Basic Research Center of Excellence for Ecological Security and Green Development, Key Laboratory for City Cluster Environmental Safety and Green Development of the Ministry of Education, School of Ecology, Environment and Resources, Guangdong University of Technology, Guangzhou, 510006 P. R. China

**Keywords:** Environmental impact, Environmental impact

## Abstract

Mercury exposure poses significant threats to human health, particularly in its organic form, methylmercury (MeHg). Diet is the main pathway for human MeHg exposure, especially through seafood consumption. In this context, numerous studies have established seafood MeHg concentration datasets to assess MeHg-related health risks from seafood consumption. However, existing datasets are limited to specific regions and short-term observations, making it difficult to support continuous and dynamic assessments of global MeHg-related health risks. This study takes a bottom-up approach to construct a global seafood MeHg concentration dataset during 1995-2022. Firstly, it compiles a long-term time series marine-scale dataset of seafood MeHg concentrations, based on the reported seafood mercury concentrations from existing literature and machine learning methods. Subsequently, this study used the seafood catch volumes of each nation in different marine areas as weights to estimate the national-scale seafood MeHg concentrations. This dataset can provide essential data support for environmental impact assessment of mercury and its compounds as mentioned in Articles 12 and 19 of the Minamata Convention on Mercury.

## Background & Summary

Methylmercury (MeHg), a highly toxic mercury (Hg) compound, can significantly impair the human nervous system^1^, cardiovascular system^2,3^, and immune system^4^. These health risks would further lead to economic losses. For example, Zhang *et al*. found that global economic loss induced by MeHg-related health risks could reach $117 billion annually^5^. In this context, over 150 nations and organizations have become signatories to the Minamata Convention on Mercury, which aims to mitigate the adverse impacts of Hg and its compounds on human health and the environment^6^.

Human beings are exposed to MeHg through various pathways, including direct inhalation of anthropogenic Hg emissions and the use of Hg-containing products^3^. Diet is the primary route of human exposure to MeHg, particularly through seafood consumption^7^. For example, 87%-95% of total Hg exposure among U.S. adults is attributed to fish consumption^8^. Moreover, in coastal island nations (e.g., the Seychelles Islands), where seafood consumption is a staple in local diet, MeHg exposure levels among residents far exceed the general population^9^. Therefore, monitoring MeHg concentrations of seafoods is important. It helps assess MeHg-related health risks for populations and consequently provide scientific dietary guidelines for the public^10^.

Over the past 30 years, numerous individual studies have monitored MeHg concentrations of seafoods. Meanwhile, researchers have compiled regional seafood MeHg concentration databases based on existing research data. However, these studies have primarily focused on economically developed regions (e.g., the U.S. and Europe) and high Hg emitters (e.g., China)^10–12^. Moreover, most studies have only reported MeHg concentrations of seafoods at a single time point or over a short time period. These limitations restrict the usefulness of MeHg concentration data in two aspects. On one hand, the geographical limitations prevent a comprehensive assessment of MeHg levels of seafoods worldwide, consequently hindering a systematic evaluation of global MeHg-related health risks. On the other hand, the limited time span of the data restricts the understanding of temporal trends in MeHg concentrations of seafoods. This makes it difficult to effectively identify and provide early warnings for high-risk regions dynamically.

To address the limitations of existing studies, this study constructed a global dataset of seafood MeHg concentrations during 1995-2022. For most nations, data on MeHg concentrations of their seafoods from existing research records are extremely limited. Thus, this dataset is constructed using a bottom-up approach. Specifically, we first compiled a dataset of seafood MeHg concentrations from various global marine areas during 1995-2022. Subsequently, the national MeHg concentrations of seafoods were estimated based on national fishing structure of seafoods (see details in Methods). This dataset provides an important data foundation for the assessment of environmental impacts of Hg and its compounds as pointed out in Articles 12 and 19 of the Minamata Convention on Mercury^6^. It can facilitate the development and adjustment of national food safety standards and public health policies.

## Methods

**Fig. 1:**
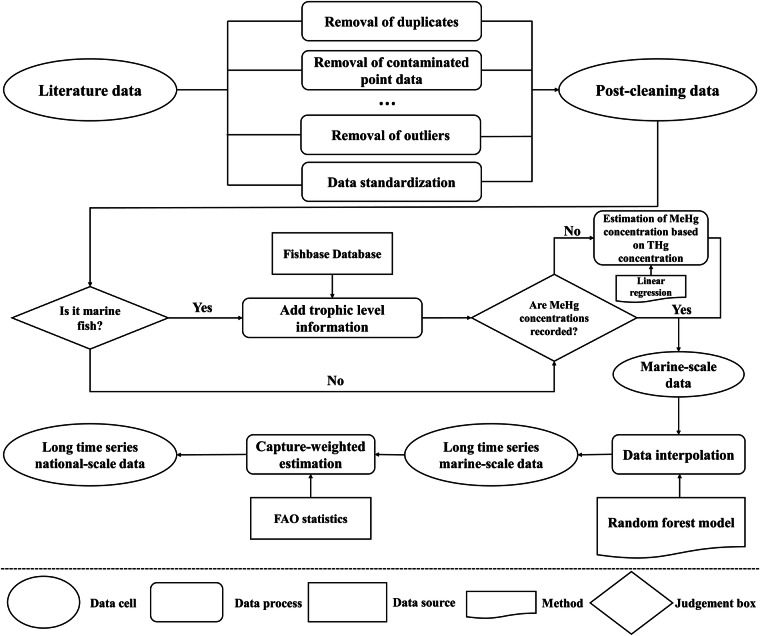
Framework for constructing global seafood MeHg concentration dataset.

The process of constructing global seafood MeHg concentration dataset is shown in Fig. 1. First, we constructed a long-term time series dataset of seafood MeHg concentrations from all marine areas based on existing literature and statistical methods. Subsequently, we estimated the national seafood MeHg concentrations based on fishing structure. The data sources of this study are shown in Table 1.

**Table 1:** Data sources of this study.

Contents	Data sources
Trophic levels of marine fishes	https://www.fishbase.se/search.php (available in January, 2025)
Seafood capture data	https://www.fao.org/fishery/en/collection/capture?lang=en (available in January, 2025)
FAO fishing areas	https://www.fao.org/fishery/en/area/search (available in January, 2025)

### Compiling long-term time series marine-scale datasets of seafood MeHg concentrations

The construction of long-term time series datasets of seafood MeHg concentrations at the marine scale primarily involved the following steps: data collection, standardization, and classification; estimation of seafood MeHg concentrations at the marine scale; and imputation of missing data.

#### Collection of seafood MeHg concentrations

The total Hg (THg) and MeHg concentrations of seafoods were retrieved from peer-reviewed studies published between 1950 and 2024 in the Web of Science database. The literature search employed a Boolean combination of the following phrases: (1) “mercury” and (2) “fish OR seafood OR crustaceans OR mollusks”. Subsequently, we applied the following criteria to screen the literature:

1. Removing duplicate data records, such as duplicate original research data in review articles^10^.
2. Excluding data from sampling sites affected by point source pollution, because MeHg concentrations in these areas are typically higher and potentially lead to an overestimation of regional MeHg concentration in seafoods^5,10,13,14^. Only data points explicitly identified as originating from polluted areas were excluded, rather than entire studies. Polluted sites were defined based on descriptions in the source literature, such as proximity to industrial activities (e.g., in the influence range of gold mines, power plants, or chemical factories), evidence of environmental contamination (e.g., abnormal Hg concentrations in local biota), or locations in known high-risk zones (e.g., areas with a history of industrial discharge or mining activities). When such information was not clearly provided, data were retained to avoid unnecessary exclusions.
3. Excluding MeHg concentrations from non-muscle tissues, because MeHg primarily accumulates in muscle tissue and data from other tissues may underestimate MeHg concentrations of seafoods^10^.
4. Removing data with unidentified marine areas.
5. Removing data with unspecified weight type (i.e., dry weight or wet weight).
6. Removing data below the detection limit.
7. Removing 5% of outliers from the data using the interquartile range rule^15^.

#### Standardization of seafood MeHg concentrations

To standardize Hg concentration data, we extracted and transformed the indicators described in Table S1 (see supplementary xlsx file). Specifically, we converted all concentration units to nanograms per gram (ng/g) and converted dry weight to wet weight, assuming a water content of 80%^10,13^, as shown in Eq. 1^16^. For studies that only reported concentration means but not standard deviations, we assumed a standard deviation of 65% of the mean^14^. In addition, for studies that did not explicitly specify the sampling year, we assumed the actual sampling year to be two years prior to the publication year^17^. In cases where research only provides seafood MeHg concentrations for a specific time period, this study assumes that the seafood MeHg concentration in each year of that period is equal to the reported value. Finally, based on the sampling location information provided in the literature, we categorized the data according to the fishing areas defined by the Food and Agriculture Organization of the United Nations (FAO). The FAO fishing areas include Arctic Sea, Atlantic Antarctic, Atlantic Eastern Central, Atlantic Northeast, Atlantic Northwest, Atlantic Southeast, Atlantic Southwest, Atlantic Western Central, Indian Ocean Antarctic, Indian Ocean Eastern, Indian Ocean Western, Mediterranean and Black Sea, Pacific Antarctic, Pacific Eastern Central, Pacific Northeast, Pacific Northwest, Pacific Southeast, Pacific Southwest, and Pacific Western Central.

1 $${WW}=\left(1-0.8\right)\ast {DW}$$

The notations *WW* and *DW* represent the wet weight and dry weight, respectively.

#### Classification of seafoods

There are significant differences in MeHg concentrations among different seafoods. Generally, MeHg concentrations of marine fishes are higher than those of other seafoods, particularly in predatory fishes^18,19^. Consequently, we classified seafoods into two groups: marine fishes and other seafoods. This classification facilitates a more detailed analysis and understanding of MeHg concentrations in different seafoods. Moreover, the vast diversity of seafood species hinders the construction of long-term MeHg concentration datasets for each individual species based on existing research. The trophic level is a crucial factor influencing seafood MeHg concentrations, with higher trophic levels generally exhibiting higher MeHg concentrations^20,21^. Following the approach by Zhang *et al*.^5^, we classified marine fishes based on their trophic levels and constructed MeHg concentration datasets for different trophic level categories. Trophic levels of marine fishes were obtained from the Fishbase database (Table 1). Based on their trophic levels, we classified marine fishes into four categories: Level 1 (1.5-2.5), Level 2 (2.5-3.5), Level 3 (3.5-4.5), and Level 4 (4.5-5.5)^5^. For other seafoods, due to the lack of corresponding trophic levels in existing databases (e.g., Fishbase and Sealife databases), we categorized them into a single category of “seafoods (excluding marine fishes)”.

#### Estimation of seafood MeHg concentrations at the marine scale

In most studies, only THg concentrations of seafoods were reported. To address the lack of direct MeHg data, we followed previous studies by converting THg to MeHg using a regression relationship derived from available data where both THg and MeHg values were reported^13,14^ (Eqs. 2 and 3). To mitigate the uncertainties inherent in using predicted values rather than direct measurements, we employed a Monte Carlo simulation, running it 10,000 times to convert the THg data from studies that only reported THg values into corresponding MeHg estimates. The resulting mean and standard deviation from these simulations represent the uncertainty range of the MeHg data. Notably, most studies report the mean concentrations of all samples, rather than the concentrations of individual samples. Therefore, we recorded the sample size for each study and used these sample sizes as weights to estimate the mean concentrations of seafoods in each marine area (Eq. 4). For studies that did not provide sample size information, we assumed a sample size of 1 to reduce potential biases that such data might introduce when estimating the overall mean.

2 $${{MeHg}}_{{MFS}}={e}^{\mathrm{ln}\left({{THg}}_{{MFS}}\right)-0.23}$$

3 $${{MeHg}}_{{OTS}}={e}^{0.94\ast \mathrm{ln}\left({{THg}}_{{OTS}}\right)-0.47}$$

4 $${C}_{{ljt}}=\frac{\mathop{\sum }\limits_{i=1}^{n}{C}_{{lijt}}\ast {m}_{{lijt}}}{\mathop{\sum }\limits_{i=1}^{n}{m}_{{lijt}}}$$

The notations *MeHg*_*MFS*_ and *MeHg*_*OTS*_ represent the MeHg concentrations of marine fishes and other seafoods, respectively, while *THg*_*MFS*_ and *THg*_*OTS*_ represent the THg concentrations of marine fishes and other seafoods, respectively; *C*_*ljt*_ represents the sample-weighted MeHg concentration of seafood *l* in marine area *j* at time *t*, while *C*_*lijt*_ represents the MeHg concentration of seafood *l* in marine area *j* in study *i* at time *t*; *n* represents the total number of studies; and *m*_*lijt*_ represents the sample size of seafood *l* in marine area *j* in study *i* at time *t*.

#### Imputation of missing concentration values

**Fig. 2:**
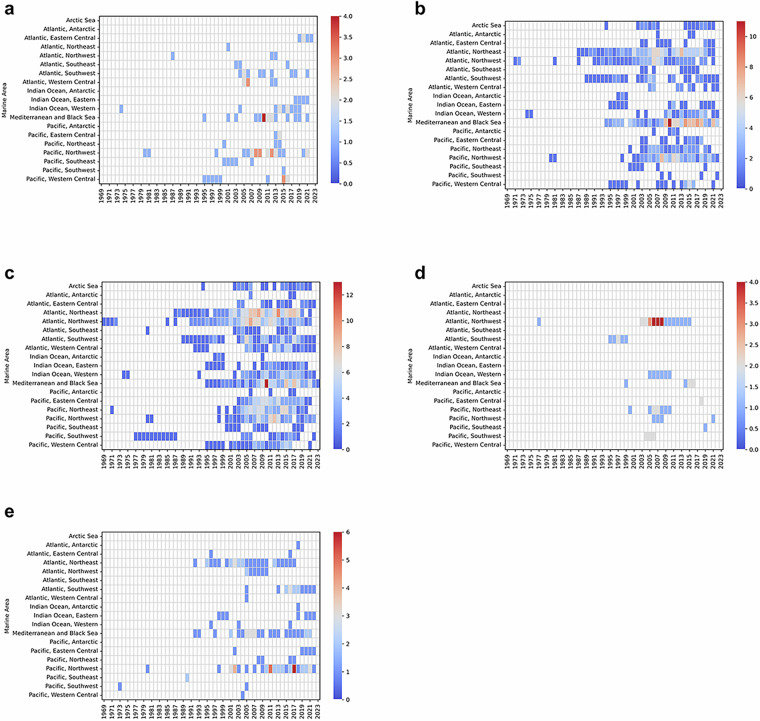
Temporal distribution of studies on seafood MeHg concentrations in different marine areas. (**a–d**) represent the number of studies on Level 1, Level 2, Level 3 and Level 4 marine fishes respectively, while (**e**) represents the number of studies on seafoods (excluding marine fishes).

In existing studies, seafood MeHg concentrations are incomplete in certain years, and data prior to 1995 are particularly scarce (Fig. 2). Therefore, the long-term time series of seafood MeHg concentrations constructed in this study starts from 1995. We employed the random forest algorithm from machine learning techniques to impute the missing values. The random forest algorithm has been widely applied in the field of data interpolation^22–25^. Due to its outstanding non-linear processing capability, the random forest algorithm shows better performance in predicting complex time series data compared to traditional linear interpolation methods. In this study, we used the “RandomForestRegressor” module in the “sklearn.ensemble” package of Python 3.11 to implement this algorithm. We randomly divided the data into a training set and a test set with a ratio of 90% and 10%, respectively^22,23^. The training set was used for model construction and training, while the test set was used to evaluate the prediction accuracy of the model. The detailed implementation processes can refer to the source code in the Zenodo^26^.

The robustness of the random forest simulation results will decrease when faced with severe data gaps. From the perspective of data availability, MeHg concentrations of Level 2 and Level 3 marine fishes are relatively complete (Fig. 2), allowing for direct interpolation using the random forest method. However, there is a significant lack of data on MeHg concentrations in Level 1 and Level 4 marine fishes, as well as other seafoods (Fig. 2). Given the significant bioaccumulation effect of MeHg in the marine food web, there is a correlation between different seafoods^27,28^. Based on this background, this study employed the random forest method to investigate their relationships with the MeHg concentrations in Level 2 and Level 3 marine fishes and utilized these relationships to impute missing values.

### Compiling long-term time series national-scale datasets of seafood MeHg concentrations

We used the seafood catch volumes from different marine areas as weights to calculate the national MeHg concentrations of seafoods (Eq. 5). The seafood catch volumes were collected from FAO (Table 1). In this study, we adopted the International Standard Statistical Classification of Aquatic Animals and Plants (ISSCAAP). Specifically, the “Marine Fishes” category was used to represent marine fishes, and the “Crustaceans” and “Molluscs” categories were used to represent other seafoods. Moreover, we classified the marine fish catch volumes based on their trophic levels into the four categories mentioned previously: Level 1 (1.5-2.5), Level 2 (2.5-3.5), Level 3 (3.5-4.5), and Level 4 (4.5-5.5). The seafood catch volumes for certain nations were recorded as zero in certain years. Following Zhang *et al*.^5^, we imputed missing seafood MeHg concentrations using the geometric mean of seafood MeHg concentrations from the corresponding continent of each nation. If MeHg concentrations for the whole continent were unavailable, the geometric mean of global MeHg concentration data was used as a replacement.

5 $${C}_{{lmt}}=\frac{\mathop{\sum }\limits_{j=1}^{n}{C}_{{ljt}}\ast {F}_{{lmjt}}}{\mathop{\sum }\limits_{j=1}^{n}{F}_{{lmjt}}}$$

The notation *C*_*lmt*_ denotes the MeHg concentration of seafood *l* in nation *m* at time *t*; *C*_*ljt*_ represents the sample-weighted MeHg concentration of seafood *l* in marine area *j* at time *t*; *F*_*lmjt*_ represents the quantity of seafood *l* caught by nation *m* in marine area *j* at time *t*; and *n* denotes the number of marine areas.

## Data Records

The raw data involved in this study, as well as the compiled MeHg concentration data of seafoods from different marine regions and nations, have been uploaded to Zenodo in XLSX and CSV formats^26^. The raw data are stored in XLSX files named “Raw_species.xlsx”, where “species” represents the seafood type, with “FISH” for marine fishes and “OSE” for other seafoods. The processed data used as input for the model are stored in CSV files named “Model_species.csv”. Similarly, “species” represents the seafood type, where “FISH” represents marine fishes, and “OSE” represents other seafoods. The capture structures of seafoods are stored in CSV files named “Category_year_capture.csv”, where category represents the seafood category, with values including Level 1, Level 2, Level 3, Level 4, and OSE. The MeHg concentrations of seafoods from different marine areas and nations are stored in CSV files named “scale_species_statistics.csv”, where “scale” indicates the data scale (i.e., “Marine” for marine regions and “Nation” for countries), “species” represents the seafood category, and “statistics” represents the type of data provided (i.e., “mean” for mean values and “std” for standard deviations of MeHg concentrations). The file “List of literature.xlsx” provides the names of the references from which the concentration data were collected, and “Nation_ID.xlsx” contains the ISO3 codes for each nation. The detailed descriptions of the column parameters in the dataset are provided in Table S1 (see supplementary xlsx file).

## Technical Validation

**Fig. 3:**
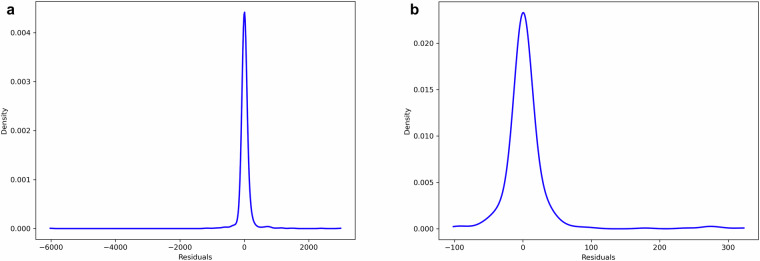
Kernel density estimate of residuals for model evaluation. (**a**) and (**b**) represent the kernel density estimates of residuals for marine fishes and seafoods (excluding marine fishes), respectively.

This study conducted a residual analysis to validate the THg to MeHg conversions. The residual analysis for marine fishes demonstrates that the model provides robust predictions of MeHg concentrations. The residual density plot shows a sharp peak centered around zero (Fig. 3a), indicating that most prediction errors are minimal. Moreover, the long but low-density tails on both sides of the distribution reveal that only a small fraction of the predictions exhibit larger errors (Fig. 3a). Overall, the analysis supports the reliability of the model in predicting MeHg concentrations for marine fishes.

For seafoods (excluding marine fishes), the residual analysis also shows that the model predictions are reliable. The residual distribution is centered around zero, indicating that most prediction errors are small (Fig. 3b). Unlike marine fishes, the tails in this distribution are shorter (Fig. 3b), suggesting fewer extreme prediction errors. However, some residuals deviate slightly to the right (Fig. 3b), indicating a potential slight overestimation in certain cases. Overall, the analysis confirms that the model is robust for predicting MeHg concentrations in seafoods (excluding marine fishes).

This study estimated MeHg concentrations for different trophic levels of marine fishes and seafoods (excluding marine fishes) in various marine areas and nations. However, these estimates cannot be directly compared with species-specific observation data reported in the existing literature. The global seafood MeHg concentration datasets are subject to three uncertainties:

1. The variation in MeHg concentration measurement methods employed by different studies may introduce methodological biases into data analysis. Specifically, the determination of MeHg requires the combination of Hg determination and Hg speciation analysis. Hg determination methods include, but are not limited to, cold vapour atomic absorption spectrometry (CVAAS), cold vapour atomic fluorescence spectrometry (CVAFS), and inductively coupled plasma-mass spectrometry (ICP-MS), which differ in sensitivity, detection limit, and accuracy^17^. Moreover, Hg speciation analysis techniques, such as gas chromatography (GC), liquid chromatography (LC), and ionic chromatography (IC), also influence the final determination results^19^. These methodological differences may lead to significant discrepancies in reported MeHg concentrations for the same sample across different studies, thereby affecting data integration.
2. The seafood catch data provided by the FAO does not include non-commercial fisheries, such as subsistence fisheries, recreational fisheries, and illegal fishing, leading to an underestimation of actual catch volumes^29^. As catch is a crucial weighting factor in estimating national MeHg concentrations of seafoods, the lack of data on these fisheries directly affects the accuracy of MeHg concentration estimates.
3. There were missing values in seafood MeHg concentrations in certain years. This study used random forest algorithms to estimate these missing values. During the model construction process, environmental factors that affect seafood MeHg concentrations (e.g., MeHg concentrations in phytoplankton and seawater, and dissolved organic carbon concentration) could not be incorporated due to the unavailability of long-term time series data for these factors^30,31^. Consequently, the prediction accuracy of the model may be limited due to the lack of multidimensional feature data. Moreover, most existing studies focus on the measurement of THg, while measured data for MeHg remain relatively scarce. This underrepresentation of MeHg measurements introduces further uncertainty into the dataset, as the reliance on THg to MeHg conversions inherently depends on the accuracy of the regression models used.

We used 10,000 Monte Carlo simulations to estimate the uncertainties in seafood MeHg concentrations^32,33^. In the dataset, the uncertainty range of seafood MeHg concentrations was quantified using mean and standard deviation. To reduce the uncertainties presented in the dataset, efforts can be directed toward the following three aspects:

1. Developing standardized analytical protocols to unify the methods for Hg determination and speciation analysis. This measure will effectively mitigate the determination biases arising from different studies.
2. Regional collaboration and remote sensing technology should be employed to expand the scope and depth of fisheries data collection. Furthermore, a more comprehensive regional data aggregation mechanism is recommended to encourage nations to provide more complete catch data. These measures will significantly enhance the comprehensiveness and accuracy of fisheries data, thereby improving the precision of seafood MeHg concentration estimates for various nations.
3. Strengthening the long-term monitoring of relevant environmental factors (e.g., MeHg concentrations in phytoplankton and seawater, and dissolved organic carbon concentrations) and integrating these monitoring data as feature variables into the model can provide more comprehensive input data support for the model. This will contribute to improving the prediction accuracy and reliability of the model. Moreover, efforts should be made to promote the collection and reporting of MeHg concentrations in seafoods. Collaborative initiatives between research institutions and monitoring programs could facilitate MeHg data collection in underrepresented regions, further enhancing the robustness of future datasets.

## Usage Notes

The dataset compiled in this study has two potential applications. On one hand, the raw dataset contains THg and MeHg concentrations of global seafoods, supporting a diverse range of research endeavors. For example, this dataset can be used to evaluate the effectiveness of the implementation of the Minamata Convention on Mercury and to identify potential pollution sources. On the other hand, it can provide a data foundation for researchers to assess the potential impacts of MeHg on ecosystems and human health at global and regional scales.

## Supplementary information

Supplementary Table S1

## Data Availability

The data processing was performed in Microsoft Excel LTSC 16.0. Python 3.11 was used for modeling, and the related codes can be available on Zenodo^26^.
